# Differentiating glaucoma from chiasmal compression using optical coherence tomography: the macular naso-temporal ratio

**DOI:** 10.1136/bjo-2023-323529

**Published:** 2023-06-28

**Authors:** Iris Kleerekooper, Siegfried K Wagner, S Anand Trip, Gordon T Plant, Axel Petzold, Pearse A Keane, Anthony P Khawaja

**Affiliations:** 1 Department of Neuroinflammation, UCL Queen Square Institute of Neurology, University College London, London, UK; 2 Department of Neurophthalmology, Moorfields Eye Hospital NHS Foundation Trust, London, UK; 3 Dutch Expertise Centre for Neuro-ophthalmology & MS Centre, Departments of Neurology and Ophthalmology, Amsterdam UMC, Amsterdam, Netherlands; 4 Institute of Ophthalmology, University College London, London, UK; 5 NIHR Moorfields Biomedical Research Centre, Moorfields Eye Hospital NHS Foundation Trust and UCL Institute of Ophthalmology, London, UK; 6 University College London Hospitals (UCLH) NIHR Biomedical Research Centre, University College London Hospitals NHS Foundation Trust, London, UK; 7 Department of Brain Repair and Rehabilitation, Institute of Neurology, University College London, London, UK

**Keywords:** Glaucoma, Retina

## Abstract

**Background/aims:**

The analysis of visual field loss patterns is clinically useful to guide differential diagnosis of visual pathway pathology. This study investigates whether a novel index of macular atrophy patterns can discriminate between chiasmal compression and glaucoma.

**Methods:**

A retrospective series of patients with preoperative chiasmal compression, primary open-angle glaucoma (POAG) and healthy controls. Macular optical coherence tomography (OCT) images were analysed for the macular ganglion cell and inner plexiform layer (mGCIPL) thickness. The nasal hemi-macula was compared with the temporal hemi-macula to derive the macular naso-temporal ratio (mNTR). Differences between groups and diagnostic accuracy were explored with multivariable linear regression and the area under the receiver operating characteristic curve (AUC).

**Results:**

We included 111 individuals (31 with chiasmal compression, 30 with POAG and 50 healthy controls). Compared with healthy controls, the mNTR was significantly greater in POAG cases (β=0.07, 95% CI 0.03 to 0.11, p=0.001) and lower in chiasmal compression cases (β=−0.12, 95% CI −0.16 to –0.09, p<0.001), even though overall mGCIPL thickness did not discriminate between these pathologies (p=0.36). The mNTR distinguished POAG from chiasmal compression with an AUC of 95.3% (95% CI 90% to 100%). The AUCs when comparing healthy controls to POAG and chiasmal compression were 79.0% (95% CI 68% to 90%) and 89.0% (95% CI 80% to 98%), respectively.

**Conclusions:**

The mNTR can distinguish between chiasmal compression and POAG with high discrimination. This ratio may provide utility over-and-above previously reported sectoral thinning metrics. Incorporation of mNTR into the output of OCT instruments may aid earlier diagnosis of chiasmal compression.

**What is already known on this topic**

- Individuals with chiasmal compression frequently experience delayed diagnosis owing to non-specific visual disturbance at an early stage.

**What this study adds**

- This report suggests that an optical coherence tomography-based index, the macular naso-temporal ratio, can discriminate between individuals with primary open angle glaucoma and chiasmal compression.

**How this study might affect research, practice or policy**

- The macular nasotemporal ratio is a novel easy to implement metric, which can aid early detection of chiasmal compression.

## Introduction

The most relevant predictor of good visual outcome in chiasmal compressive lesions is early surgery.^1^ Visual symptoms in chiasmal compression can remain unnoticed by patients until the deficit is dense or affects central vision.^2^ The risk for delayed diagnosis and poor outcome is recognised in clinical practice and is particularly relevant for glaucoma services monitoring the visual field.^3^ Similar to patterns of visual field defects, patterns of macular atrophy on optical coherence tomography (OCT) likely convey important diagnostic clues, which can be obscured in overall retinal thickness measures.^4 5^

Individuals with chiasmal compression develop direct retrograde degeneration of the decussating fibres in the anterior visual pathway, which causes predominant atrophy of the nasal hemi-macular ganglion cell and inner plexiform layer (mGCIPL),^6–10^ giving rise to the ‘half-moon’ sign.^4^ Clinically, this is associated with the typical perimetric finding of bitemporal hemianopia. In contrast, glaucoma is generally more likely to present with visual field defects in the nasal region,^11^ often presenting as an altitudinal ‘nasal step’ and corresponding to atrophy of the temporal hemimacular field.^12^ Structural changes in inner retinal thickness, as observed with OCT, can precede perimetric defects in chiasmal compression,^2 13 14^ depending on test settings^3^ and are associated with postsurgical visual recovery.^15–19^ General limitations of perimetry include the high variability of results,^20–22^ differences in approach of examiners, learning effects and test failure due to tiredness or reduced attention (reviewed in Petzold *et al* 21).

The widespread availability of OCT in eye clinics and community optometric practices mean that macular thickness pattern analysis could potentially improve detection of chiasmal compression. In this study, a comprehensive measure of macular atrophy distribution was created by comparing the nasal and temporal mGCIPL thickness through the macular naso-temporal ratio (mNTR). The aim was to quantify the diagnostic value of the mNTR in distinguishing two common causes of optic nerve damage, chiasmal compression and glaucoma.

## Methods

### Participants

Patients with preoperative chiasmal compressive lesions and primary open-angle glaucoma (POAG) were identified retrospectively through a structured query language search of the electronic health record at Moorfields Eye Hospital NHS Foundation Trust in London, United Kingdom. More concretely, for patients with chiasmal compression, the search extracted patients (1) with the term ‘chiasmal’, ‘pituitary’ or ‘bitemporal hemianopia’ in their letters and (2) cross-referenced with those who had a previous macular Heidelberg OCT scan. All clinical records and images were then manually validated by clinicians for cases with MRI-confirmed chiasmal compression and available preoperative macular OCT scans of sufficient quality. For POAG cases, the search extracted patients (1) with the term ‘POAG’ or ‘glaucoma’ in their clinical letters, (2) attending glaucoma clinics at MEH and (3) cross-referenced with those who had a previous macular Heidelberg OCT scan. Clinical records and images were then manually validated by clinicians in reverse date order (ie, most recent search results were validated first) for those who had a diagnosis of POAG made by a consultant ophthalmologist specialising in glaucoma, until 30 suitable cases were included. We excluded patients with any ocular or retinal disease other than POAG, or any central nervous system lesions (such as stroke or neoplasm) outside of the chiasmal region. All healthy control participants were examined and scanned by one coauthor (AP) and part of previously reported cohort.^23 24^

### Ethics

This study of retrospective routinely collected data was approved by the Moorfields Eye Hospital Institutional Review Board (Health Research Authority reference: 20/HRA/2158) and the analysis of control data by the Amsterdam University Medical Centre Institutional Review Board (reference: 2010/336). All control subjects gave written informed consent.

### OCT scan acquisition

OCT scans carried out as part of routine clinical care were retrospectively identified for patients with POAG and chiasmal compression. Macular volume scans of both eyes obtained with Spectralis SD-OCT (Heidelberg Engineering, Heidelberg, Germany) that passed OSCAR-IB quality control criteria were included.^25^

For healthy controls, the OCT measurements were performed prospectively with a Spectralis SD-OCT with the eye-tracking function enabled, using acquisition software V.6.7.13.0. Macular volume scans (1024 A-scans, 37 B-scans volume=15×15°, automatic real-time function = 25) centred on the fovea with the high-resolution setting enabled were performed, with subsequent scans performed on follow-up mode.

### Image analysis

All B-scans were auto-segmented using Heidelberg Eye Explorer (V.6.15.7.0), followed by manual correction where required. Presence of microcystic macular oedema (MMO) was determined visually as described.^26^ The macular ganglion cell layer and inner plexiform layer were segmented. Compound mGCIPL thickness was calculated. Mean layer thicknesses were computed within a 4×4 square grid centred on the fovea. The thicknesses in the inferior-nasal (IN), superior-nasal (SN), inferior-temporal (IT) and superior-temporal (ST) quadrants were combined to derive the ‘global macular thickness’, while the two nasal quadrants comprised the ‘nasal hemi-macular thickness’ and the two temporal quadrants comprised the ‘temporal hemi-macular thickness’. The mNTR was created by dividing the nasal macular thickness by the temporal macular thickness. Atrophy occurring exclusively or to a greater extent nasally would be associated with a reduced mNTR, while atrophy occurring exclusively or to a greater extent temporally would be associated with an increased mNTR. This is analogous to the nasal temporal ratio, which already exists for the peripapillary retinal nerve fibre layer in the ‘neuro’ protocol of the Heidelberg Spectralis. In addition, the NI/NS and the NI/TS ratios were calculated, as chiasmal compression is known to preferentially affect the NI quadrant first.^27^

### Statistical analysis

Data were analysed using R and Rstudio (RStudio Team 2021, http://www.rstudio.com/). Continuous variables were described by means and SD, and categorical variables by counts and percentages. Distributions of continuous variables and dichotomous variables across diagnosis groups (POAG, chiasmal compression and healthy controls) were inspected visually and tested with the Kruskal-Wallis and Fisher exact tests, respectively. We corrected for multiple comparisons of the post hoc analysis (Dunn Test) by adjusting p values using the Benjamini-Hochberg method. To investigate whether the mNTRs differed between the two diagnostic and control groups, a multivariable linear regression analysis was performed adjusted for age, sex and overall mGCIPL thickness. Performance of the mNTR and the overall mGCIPL thickness in discriminating glaucoma and pituitary lesions were analysed by plotting receiver operating characteristic (ROC) curves and calculating the associated area under the curve (AUC) and associated 95% CIs using bootstrapping methods. AUC of different ROC curves was compared with the DeLong method. Optimal cut-off values were calculated using the Youden Index. To account for inter-eye correlations, the mean value of the two eyes for each subject was calculated and analysed.^28^ A sensitivity analysis was performed on the eyes with the thinner and thicker mGCIPL overall thickness separately. Statistical significance threshold was set at p<0.050.

## Results

### Participants

**Figure 1:**
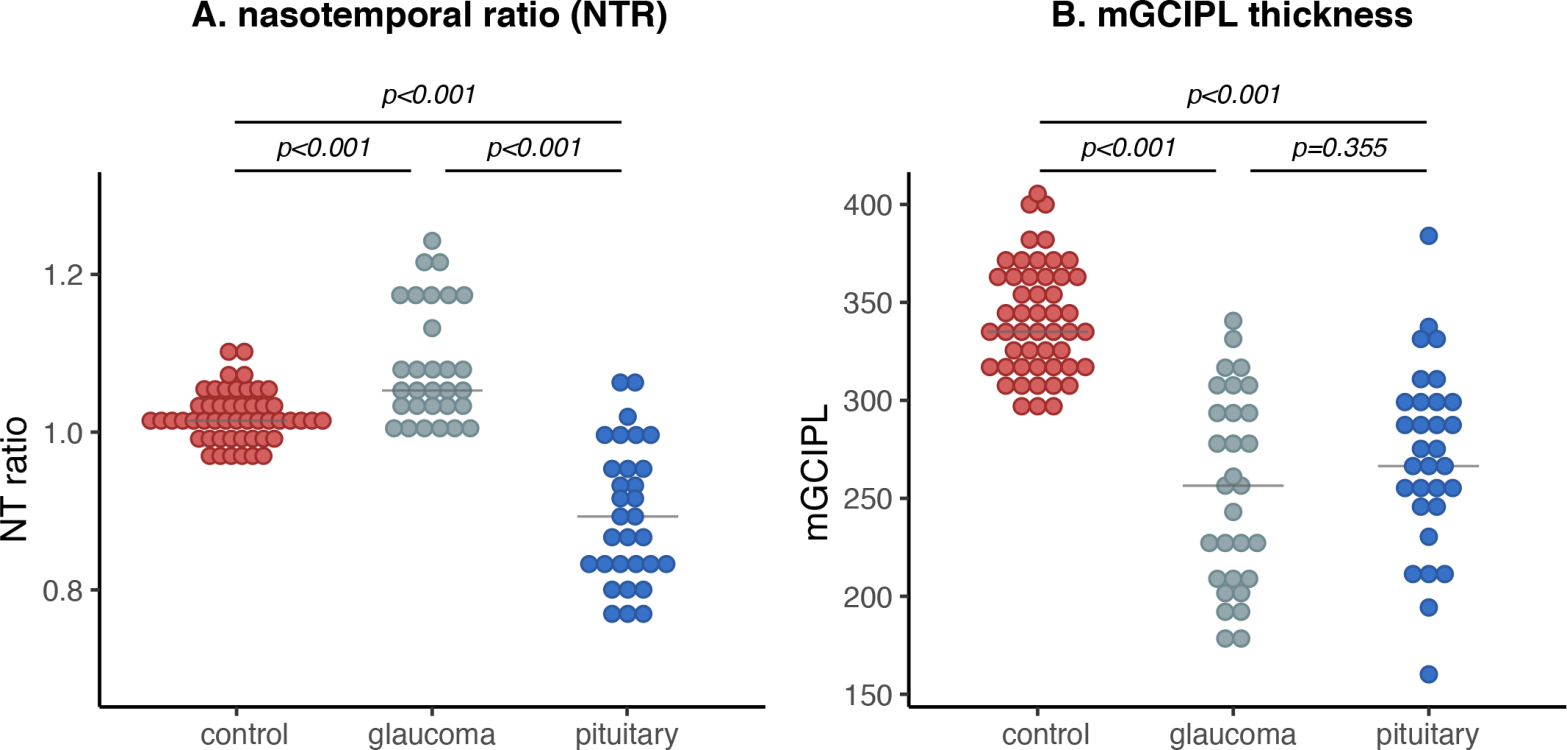
Dot plots showing macular naso-temporal ratio (mNTR) and macular ganglion cell and inner plexiform layer (mGCIPL) thickness across groups in (A) and (B) respectively. P values represent results of post-hoc evaluation of intergroup differences with the Dunn-test, adjusted for multiple comparisons through the Benjamini-Hochberg method. Horizontal lines represent medians.

Table 1 shows the baseline characteristics of the included cohort. A total of 31 subjects with preoperative chiasmal tumours, 30 patients with POAG and 50 healthy controls were included (online supplemental figure). The causes of chiasmal compression were pituitary macroadenoma (n=24), suprasellar meningioma (n=2), unspecified suprasellar mass (n=2), chiasmal glioma (n=2) and craniopharyngioma (n=1). Sex did not statistically differ between groups. All but one of the patients with chiasmal compression went on to have surgical resection of the compressing lesion, which occurred between 1 month and 4 years after the analysed OCT scan was acquired (median: 5 months). Age differed significantly between the three groups (all p<0.050). mGCIPL thickness was significantly lower in glaucoma and pituitary lesion compared with healthy controls (both p<0.001). However, there was no significant difference in overall mGCIPL thickness between subjects with POAG and chiasmal compression (p=0.36) (figure 1A and 1B). As far as these data were available (for 31 chiasmal compression cases and 17 POAG cases), visual acuity was similar for chiasmal compression cases compared with POAG, with a median of 6/6 for POAG and 6/9 for chiasmal compression. These data seem to suggest a relatively similar severity of disease in both groups.

10.1136/bjo-2023-323529.supp1 Supplementary data

**Table 1:** Baseline characteristics table

	Chiasmal compression	POAG	Control	Test P value
N	31	30	50	
Female sex, N (%)	15 (48%)	12 (40%)	14 (28%)	0.162*
Age, mean (SD)	49.5 (13.4)	62.6 (16.6)	42.4 (12.3)	<0.001†
mGCIPL thickness μm, mean (SD)	267 (48)	249 (50)	340 (28)	<0.001†
mNTR, mean (SD)	0.90 (0.09)	1.08 (0.07)	1.02 (0.03)	<0.001†
IN/SN ratio, mean (SD)	1.00 (0.06)	0.96 (0.07)	0.98 (0.04)	0.057†
IN/ST ratio, mean (SD)	0.93 (0.10)	1.03 (0.09)	1.01 (0.04)	<0.001†

*Results of the Fisher exact test.

†Results of Kruskal-Wallis test.

IN/SN, inferionasal/superionasal; IN/ST, inferionasal/superiotemporal; mNTR, macular naso-temporal ratio; POAG, primary open-angle glaucoma.

### mNTR across groups

The mNTR was significantly lower in chiasmal compression compared with POAG (p<0.001). Compared with healthy controls, the mNTR was significantly decreased for subjects with chiasmal compression and significantly increased in subjects with POAG (both p<0.001) (figure 1). The mNTR was not associated with age (r=0.00, p=0.377).

### Multivariable linear regression

Multivariable linear regression adjusted for sex, age and overall mGCIPL thickness identified that the mNTR was significantly lower for subjects with chiasmal compression (=−0.12, 95% CI −0.16 to −0.09, p<0.001) and significantly higher for subjects with POAG (=0.07, 95% CI 0.03 to 0.11, p=0.001), compared with controls. The IN/SN ratio did not significantly differ across the groups, and the IN/ST ratio was significantly reduced in chiasmal compression (=−0.08, 95% CI −0.13 to −0.04, p<0.001) but showed no associations with POAG (table 2).

**Table 2:** Multivariable linear regression analyses

	mNTR Mean both eyes		IN/SN ratio Mean both eyes		IN/ST ratio Mean both eyes	
	Est	P value	Est	P value	Est	P value
Controls	–	–	–	–	–	–
POAG	0.07	**0.001**	−0.02	0.185	0.02	0.476
Chiasmal compression	−0.12	**<0.001**	0.02	0.295	−0.08	**<0.001**
Age	−0.00	0.377	0.00	0.476	0.00	0.437
Sex	−0.01	0.986	−0.02	0.044	−0.03	0.104
Mean mGCIPL thickness μm	0.00	0.698	0.00	0.482	0.00	0.515

Multivariable linear regression analyses showing the distribution of inter-eye mean macular nasotemporal ratio (mNTR), the inferionasal/superionasal (IN/SN) ratio and inferionasal/superiotemporal (IN/ST) ratio across controls, glaucoma and chiasmal compression cases. The analysis is adjusted for age, sex and mean overall mGCIPL thickness. Significant results are shown in bold.

mGCIPL, macular ganglion cell and inner plexiform layer; POAG, primary open-angle glaucoma.

### Exploration of diagnostic accuracy

**Figure 2:**
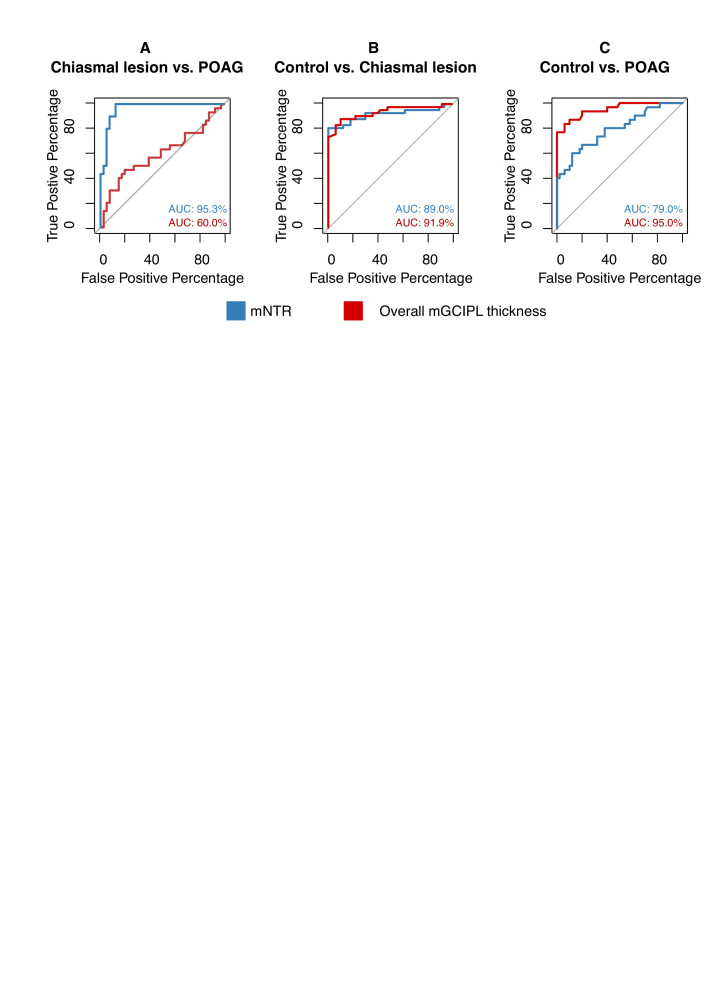
Receiver operating characteristic (ROC) curves. (A) shows the ROC curves visualising the diagnostic properties of the macular naso-temporal ratio (mNTR) (in blue) and overall macular ganglion cell and inner plexiform layer (mGCIPL) thickness (in red) when distinguishing eyes affected by chiasmal compression and primary open-angle glaucoma (POAG). (B) and (C) show the ROC curves visualising the diagnostic properties of the mNTR (in blue) and overall mGCIPL thickness (in red) when distinguishing healthy controls (HC) from eyes affected by chiasmal compression and POAG, respectively.

Given the significant results in multivariable linear regression analysis, the mNTR was taken forward for analysis with ROC curves (figure 2). The AUCs for the mNTR were 89.0% (95% CI 80 to 98%) and 79.0% (95% CI 68 to 90%) when comparing healthy controls with chiasmal compression and POAG, respectively, and 95.3% (95% CI 90% to 100%) for comparing POAG and chiasmal compression directly.

Overall mGCIPL thickness had poor performance when discriminating between POAG and chiasmal compression, with an AUC of 60.0% (95% CI 45% to 75%). mGCIPL thickness did have AUCs of 95.0% (95% CI 90% to 100%) and 91.9% (95% CI 85% to 99%) when distinguishing controls from POAG and chiasmal compression, respectively. When discriminating controls from cases with either chiasmal compression or POAG, the AUC was 94.1% (95% CI 89% to 99%).

When distinguishing chiasmal compression from POAG, an optimal diagnostic mNTR threshold of <0.99 was identified, which was associated a specificity of 100% and a sensitivity of 84%. When comparing with healthy controls, a mean mNTR threshold of <0.96 was associated with a specificity of 100% and a sensitivity of 77% in identifying chiasmal compression and an NTR threshold of >1.06 was associated with a specificity of 88% and a sensitivity of 60% in identifying glaucoma. An mGCIPL thickness of <309 µm was associated a specificity of 89% and a sensitivity of 89% for distinguishing controls from cases with either chiasmal compression or POAG (online supplemental table).

There were three patients with chiasmal compression who had no visual symptoms and had no appreciable abnormalities on Goldman visual fields. These patients had a mNTR of 0.91, 0.96 and 1.07.

### Qualitative image evaluation

**Figure 3:**
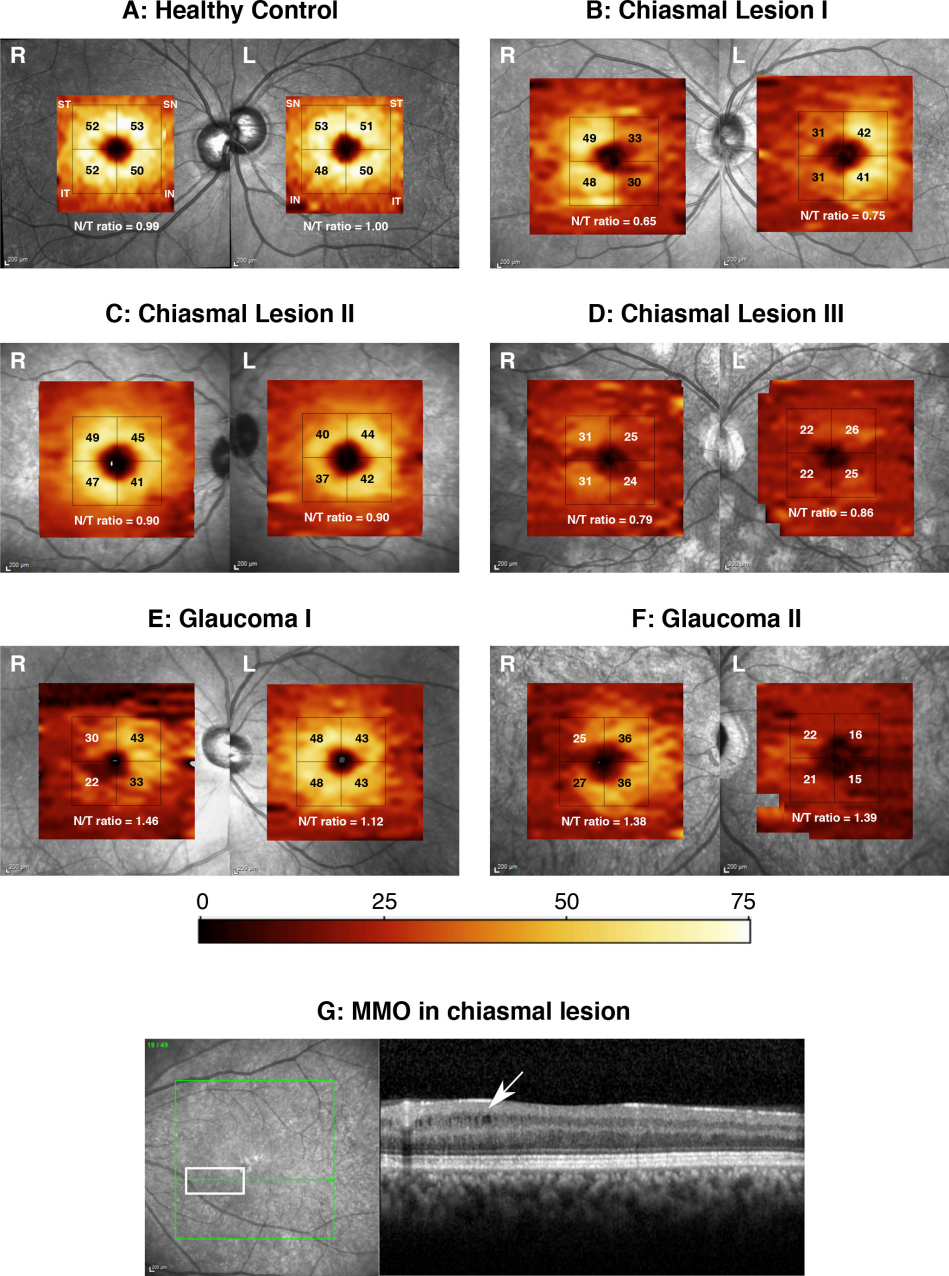
Examples of optical coherence tomography (OCT) scans in healthy controls, pituitary lesions and primary open-angle glaucoma (POAG). Example ganglion cell layer thickness maps of a typical healthy control (A) three patients with chiasmal lesions (B–D) and two patients with POAG (E) and (F) are shown. The square grid with naso-superior, naso-inferior, temporo-superior and temporo-inferior quarters is shown. The macular naso-temporal ratio (mNTR) is given in white. G shows an example of a patient with chiasmal compression showing nasal microcystic macular oedema. IN,inferior-nasal; IT, inferior-temporal; SN, superior-nasal; ST, superior-temporal.

Figure 3 shows examples of thickness maps of a typical healthy control (A), three patients with chiasmal compression (B–D) and two patients with POAG (E–F). For patients with chiasmal compression and POAG a preferential nasal and temporal atrophy pattern can be visually appreciated, respectively. These patterns with associated changes in the mNTR can be observed both for eyes that are in the early (figure 3C,E) and in the advanced stages (figure 3D,F) of disease. MMO was observed in the retinal scans of three patients with pituitary lesions (9.7%) and none of the glaucoma patients (figure 3G).

## Discussion

This study suggests that the mNTR is a novel OCT metric which may contribute to distinguishing chiasmal compression from atrophy due to POAG, as it is reduced in chiasmal compression but increased in POAG. This is based on the pattern of macular inner retinal layer atrophy,^5^ and far superior to the global mGCIPL thicknesses which were not discriminatory. These data indicate that regional macular atrophy patterns can be easily quantified and convey important diagnostic clues that are obscured by overall retinal thickness measures.

Consistent with our findings, case series have reported preferential nasal atrophy of the mGCIPL thickness in patients with chiasmal compression^29 30^ and temporal atrophy in glaucoma.^11 12^ The presence of binasal atrophy of the inner retinal layers in patients with chiasmal compression has been described qualitatively before,^29 30^ and it has been shown that nasal mGCIPL thickness is more sensitive than temporal thickness when identifying chiasmal compression.^31^ The finding of predominant nasal atrophy has been called the ‘half-moon sign’.^4^ Importantly, this pattern can also be detected in patients with POAG who later also develop compression of the chiasm. This study demonstrates that a comprehensive measure of regional retinal atrophy, being the mNTR, can distinguish chiasmal compression, POAG and controls. Retrograde degeneration due to chiasmal compression is thought to affect the inferior nasal quadrant predominantly initially, as pituitary adenomas usually compress the optic chiasm from below.^13 27^ However, atrophy in this specific quadrant, as identified through the IN/SN and the IN/ST ratios, was not or less related to pathology in this cohort. The mNTR appears to have superior diagnostic accuracy compared with analysis of reginal patterns of the retinal nerve fibre layer (RNFL), based on limited data.^32^

Although the atrophy pattern seen in glaucoma does not respect the vertical meridian, it has been described before that the temporal hemi-macula is generally more severely affected.^11 12^ This was also identified in these data, with the mNTRs being higher in eyes of patients with POAG compared with controls. The preferential temporal atrophy pattern associated with POAG is opposite to the nasal pattern seen in chiasmal compression, giving the mNTR excellent discriminative properties for distinguishing the two disorders.

Furthermore, these data showed that the overall mGCIPL thickness had a higher sensitivity for distinguishing healthy eyes from eyes affected by either chiasmal compression or POAG, but the mNTR had a greater specificity for distinguishing between POAG and chiasmal compression as causes of optic nerve damage. Comprehensive analysis of overall mGCIPL thickness and mNTR may optimise the diagnostic value of structural OCT data in optic nerve injury. Reduced overall mGCIPL thickness is highly sensitive to identifying the presence of optic nerve damage, which makes it an advantageous screening tool. Direct retrograde degeneration causes appreciable mGCIPL atrophy within 1 month of onset, as has been described in optic neuritis.^33^

There are other advantages when using retinal thickness metrics, compared with perimetry, when trying to identify chiasmal compression. OCT is quicker, more reproducible and less affected by patient-related factors as already discussed.^21^ Most importantly, mGCIPL atrophy may be more sensitive to picking up pituitary lesions compared with visual field defects, as it has been identified in a number of pituitary adenoma patients without abnormalities on perimetry.^2 13 14^ However, it should be noted that there are also rare cases where there is bitemporal hemianopia on perimetry without OCT abnormalities. Perimetry and OCT metrics should be used jointly to optimise diagnostic accuracy. Chiasmal compression cases in this cohort were identified at an eye hospital and almost all had visual symptoms with bitemporal hemianopia on perimetry. Therefore, the sensitivity and generalisability of the mNTR in early-stage patients without visual symptoms are still to be established. Patients with optic nerve changes due to chiasmal compression are sometimes initially thought to have glaucoma^34 35^ and the mNTR may be a particularly valuable diagnostic tool in this population.

While it is well-established that binasal inner retinal thinning respecting the vertical meridian occurs with chiasmal compression, we believe that the mNTR is an easily quantified metric that can be routinely presented to clinicians. In high-volume clinical scenarios which are focused on glaucoma care, for example, the qualitative finding of binasal thinning may be overlooked. An mNTR outside of a normal range can prompt a more detailed examination of the OCT as well as other examination findings.

Diagnostic accuracy for the mNTR was not perfect. For five chiasmal compression cases, the mNTR value exceeded 1.0. One of these cases had the lowest mGCIPL thickness in our cohort, suggesting severe atrophy. On the other hand, four cases had relatively less mGCIPL atrophy with a median of 320 (range: 261–381) compared with chiasmal compression cases overall (median 267). The case with the highest mNTR had no visual loss, but was diagnosed due to systemic symptoms that were caused by a prolactin secreting pituitary adenoma.

The association of MMO with structural lesions in the anterior visual pathways is well documented, and its presence should therefore prompt brain imaging.^36^ Here, MMO was observed in nearly 10% of patients with chiasmal compression, which is consistent with the 10.7% (3/28) reported before.^36^ MMO was located in the nasal area where also the most prominent mGCIPL atrophy was observed, in line with the presumed pathogenesis of MMO being a retrograde maculopathy.^6^ In glaucoma, MMO is typically absent.^26^ Dedicated brain imaging was not performed systematically in one report on the exceptional rare occurrence of MMO in individuals with glaucoma and substantial optic atrophy.^37^

Limitations of this work include the retrospective design without longitudinal follow-up, the limited sample size and the discussed selection bias towards chiasmal compression patients with visual symptoms. These findings will have to be replicated in a larger cohort to further ascertain our conclusions.

## Conclusion

Macular thickness maps can visualise retinotopic differences in atrophy patterns. The present study demonstrates that the mNTR a quantitative metric for patterns of hemi-macular atrophy which expands on earlier descriptions (‘half-moon’ sign), achieving high diagnostic accuracy for separating glaucoma from chiasmal compression. Due to the high-dimensionality of macular thickness maps, these data may provide excellent source data for the development of pattern recognition artificial intelligence algorithms that could facilitate early detection of optic nerve disease in the future.

## Data Availability

No data are available.
