# Supplementary material for: Differentiating glaucoma from chiasmal compression using optical coherence tomography: the macular naso-temporal ratio
Source: Br J Ophthalmol. 2023 Jun 28;108(5):695–701. doi: 10.1136/bjo-2023-323529 (PMC11137440; doi:10.1136/bjo-2023-323529)
Supplement: Supplementary data [file bjo-2023-323529supp001.pdf]

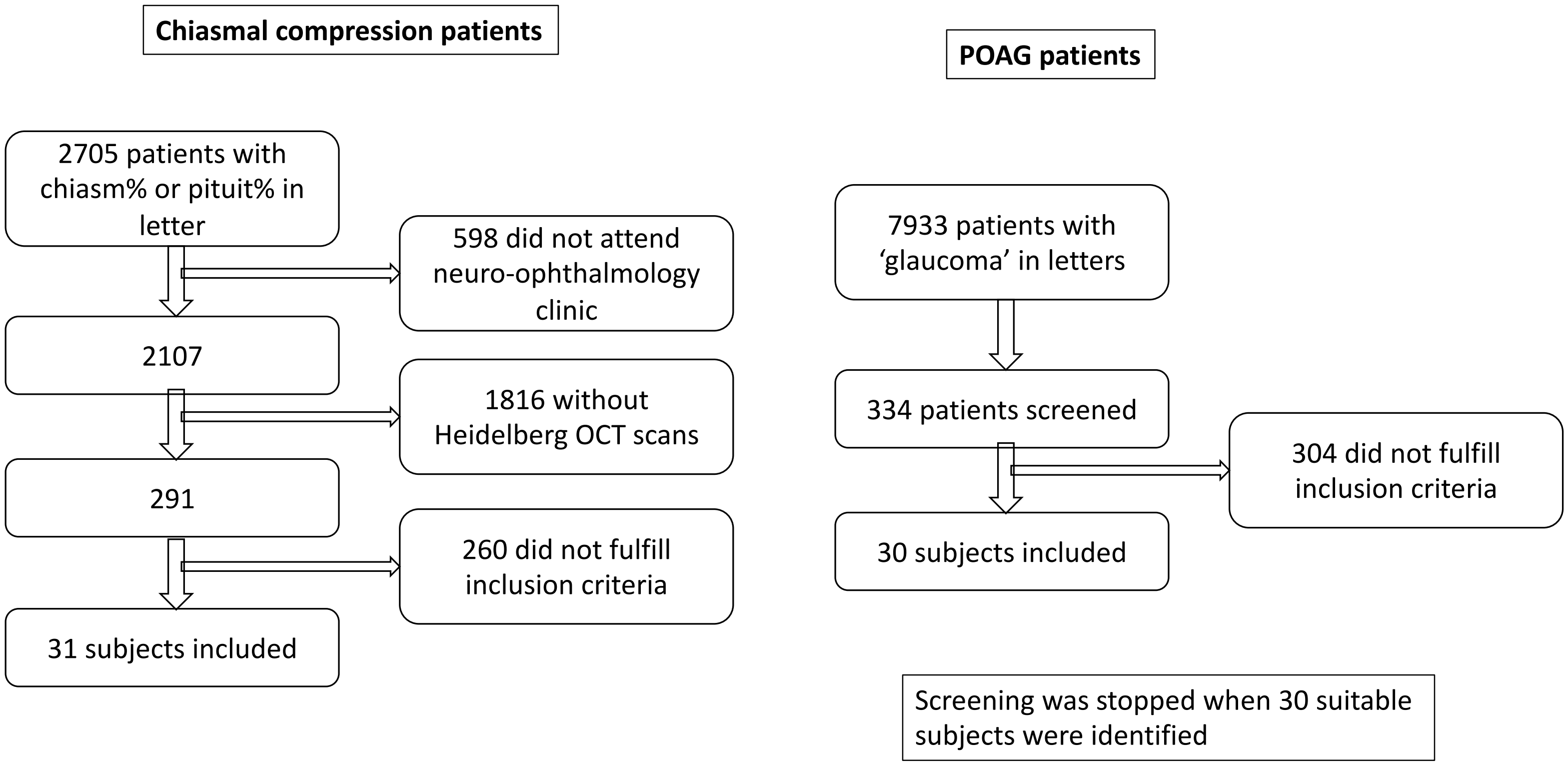

Supplementary figure: Flow chart of included patients. For chiasmal compression cases the most important exclusion factor was that the OCT scans were performed after surgical decompression (n=156), concurrent retinal disease (n=45) and insufficient OCT image quality or missing macular OCT scan (n=59). For POAG not all exclusion criteria are known, as screening was stopped when 30 suitable cases were identified.

	mNTR			mGCIPL		
	Threshold	Sensitivity	Specificity	Threshold	Sensitivity	Specificity
HC vs. chiasmal lesions	<0.96	77%	100%	<308.8	87%	90%
HC vs. POAG	>1.06	60%	88%	<301.5	83%	94%
POAG vs. chiasmal lesions	<0.99	84%	100%	<237.8	81%	47%

Supplementary table: Optimal diagnostic thresholds with associated specificity and sensitivity. Thresholds are identified through the Youden index. mNTR = macular naso-temporal ratio. mGCIPL = macular ganglion cell / inner plexiform layer. HC = healthy controls. POAG = primary open angle glaucoma.
